# Supplementary material for: SyNDI: synchronous network data integration framework
Source: BMC Bioinformatics. 2018 Nov 6;19:403. doi: 10.1186/s12859-018-2426-5 (PMC6219086; doi:10.1186/s12859-018-2426-5)
Supplement: Supplementary file 5 — Files of the example presented in Additional file 4. This additional file is a zip package that contains all files used in Additional file 4. Detailed descriptions of each of these files are presented in a README file included in this zip package. (GZ 30411 kb) [file 12859_2018_2426_MOESM5_ESM.gz › SyNDITools/Meme2Fimo/meme_4.9.0_4.tar.gz!/meme_4.9.0/doc/select-star-species.html]

select-star-species


> # select-star-species
>
> **Usage:**
>
> > `select-star-species
> > [--dist-matrix] <subset size> <tree file>`
>
> **Description:**
>
> > Given a phylogentic tree, this program attempts
> > to find the subset of species whose subtree is "closest" to a star
> > topology. By "closest" we mean that the pairwise distances between
> > distinct species are nearly equal, but are also as near as possible to
> > the average pairwise distance for the complete tree.
>
> **Algorithm:**
>
> > *Inputs:
> > > m: size of desired subset   
> > > T: phylogentic tree
> >
> > S ← List of species in T   
> > n ← size(S)   
> > **if** (n **choose** m) > 1e5 **then exit**   
> > M ← pairwise distance matrix for T   
> > μ_M_ ← mean pairwise distance for M   
> > C ← List of all subsets of S of size m   
> > best\_score ← max\_float   
> > best\_subset ← ø   
> > **for each** s **in** C
> > > score ← score(M, μ_M_, s)   
> > > **if** score < best\_score **then**
> > > > best\_score ← score   
> > > > best\_subset ← s
> >
> > **print** best\_subset   
> >   
> > score(M, μ_M_, s) ← Var(s_M_) / μ_M_
> >   
> > Var(s_M_) ← ∑ _a, b ∈ s, a ≠ b_ (M[a,
> > b] - μ_s_)^2^*
>
> **Input:**
>
> > A file containing a phylogentic tree in Newick
> > format.
>
> **Output:**
>
> > The list of species in the subset, one species
> > per line, is sent to the standard output.
>
> **Options:**
>
> > `--dist-matrix` - This option causes
> > the matrix of interspecies distances for the full tree to be written to
> > the standard output.
